# A Change in Configuration of the Calmodulin-KCNQ Channel Complex Underlies Ca^2+^-Dependent Modulation of KCNQ Channel Activity

**DOI:** 10.1371/journal.pone.0082290

**Published:** 2013-12-09

**Authors:** Anastasia Kosenko, Naoto Hoshi

**Affiliations:** Department of Pharmacology, University of California Irvine, Irvine, California, United States of America; University of Texas Health Science Center, United States of America

## Abstract

All subtypes of KCNQ channel subunits (KCNQ1-5) require calmodulin as a co-factor for functional channels. It has been demonstrated that calmodulin plays a critical role in KCNQ channel trafficking as well as calcium-mediated current modulation. However, how calcium-bound calmodulin suppresses the M-current is not well understood. In this study, we investigated the molecular mechanism of KCNQ2 current suppression mediated by calcium-bound calmodulin. We show that calcium induced slow calmodulin dissociation from the KCNQ2 channel subunit. In contrast, in homomeric KCNQ3 channels, calcium facilitated calmodulin binding. We demonstrate that this difference in calmodulin binding was due to the unique cysteine residue in the KCNQ2 subunit at aa 527 in Helix B, which corresponds to an arginine residue in other KCNQ subunits including KCNQ3. In addition, a KCNQ2 channel associated protein AKAP79/150 (79 for human, 150 for rodent orthologs) also preferentially bound calcium-bound calmodulin. Therefore, the KCNQ2 channel complex was able to retain calcium-bound calmodulin either through the AKPA79/150 or KCNQ3 subunit. Functionally, increasing intracellular calcium by ionomycin suppressed currents generated by KCNQ2, KCNQ2(C527R) or heteromeric KCNQ2/KCNQ3 channels to an equivalent extent. This suggests that a change in the binding configuration, rather than dissociation of calmodulin, is responsible for KCNQ current suppression. Furthermore, we demonstrate that KCNQ current suppression was accompanied by reduced KCNQ affinity toward phosphatidylinositol 4,5-bisphosphate (PIP2) when assessed by a voltage-sensitive phosphatase, Ci-VSP. These results suggest that a rise in intracellular calcium induces a change in the configuration of CaM-KCNQ binding, which leads to the reduction of KCNQ affinity for PIP2 and subsequent current suppression.

## Introduction

The M-current is a non-inactivating sub-threshold potassium current that regulates spike frequency adaptation and interspike frequency [1], [2], [3]. Voltage-gated M-type potassium channels are composed of various subunit combinations of four subtypes from the KCNQ gene family (KCNQ2, 3, 4 and 5). The KCNQ2 subunit has been widely used as a prototypical subunit to understand regulatory mechanisms of the M-type channel. Accordingly, calmodulin (CaM) was first identified as a co-factor of the KCNQ2 subunit [4], [5]. It has been demonstrated that CaM plays critical roles in KCNQ channel trafficking [6] as well as in channel function [7]. In addition, calcium-bound CaM, holoCaM, has been demonstrated to mediate bradykinin-induced suppression of KCNQ2/3 currents [8]. However, despite the established role of CaM as a calcium sensor, the molecular mechanism of KCNQ2 current suppression mediated by holoCaM is not well understood. In contrast to the general consensus regarding the stable association between apoCaM and the KCNQ2 subunit, contradicting results have been reported by several groups regarding the association of holoCaM with KCNQ2 and other KCNQ channel subunits [4], [5], [8], [9], [10]. Recently, the crystal structure of holoCaM and the distal CaM binding domain (Helix B) of KCNQ4 was solved [11]. This clearly shows that calcium-bound CaM can bind KCNQ channels in certain conditions.

Phosphatidylinositol 4,5-bisphosphate (PIP2) is another essential co-factor for KCNQ channels [12], [13]. Various ion channels and transporters have been shown to require PIP2 in order to function properly [14]. Not surprisingly, PIP2 has been identified as a signal mediator during muscarinic suppression of the M-current [15], [16]. We have recently demonstrated an alternative synergistic pathway, in which muscarinic stimulation reduces KCNQ2 affinity toward PIP2 via CaM dissociation from the channel complex [17].

In this report, we examined calcium effects on CaM-KCNQ2 channel interaction and channel activity. We show that holoCaM dissociated from the KCNQ2 channel due to a unique cysteine residue in the KCNQ2 subunit. A scaffold protein AKAP79/150, anchored to KCNQ2 [18], selectively bound holoCaM, functioning as an acceptor for CaM that dissociated from the KCNQ2 subunit after calcium increase. We also demonstrate that elevation of intracellular calcium reduced KCNQ2 affinity toward PIP2, as evaluated by a voltage sensitive PIP2 depleting phosphatase, Ci-VSP [19]. These results suggest that an increase in intracellular calcium suppresses KCNQ2 current by reducing PIP2 affinity due to the change in CaM-KCNQ2 configuration.

## Experimental Procedures

### Reagents and Expression Plasmids

Anti-FLAG antibody-conjugated resin and horseradish peroxidase (HRP) conjugated anti-FLAG antibody were purchased from Sigma-Aldrich (St Louis, MO, USA). Anti-AKAP150 antibody was obtained from Dr. John D. Scott (University of Washington). Anti-V5 epitope monoclonal antibody and mammalian expression vectors (pZeoSV, pcDNA3.1, pcDNA3.1/V5) were purchased from Life Technologies (Carlsbad, CA, USA). Mammalian expression constructs for the FLAG-tagged AKAP79, 3xFLAG-tagged rat KCNQ2 [18], and V5-tagged CaM [17] have been described. For the AKAP150-HIS6 construct expressed in *E. coli*, the coding sequence of AKAP150 was subcloned into pET-30b expression vector (Merck KGaA, Darmstadt, Germany). The CFP-PH construct was obtained from Dr. Tobias Meyer (Stanford University) through Addgene. Ci-VSP was obtained from Dr. Yasushi Okamura (Osaka University, Japan). CaM(4DA) mutant (D20A/D56A/D93A/D129A) [8] and KCNQ2(C527R) constructs were generated by QuikChange II XL site-directed mutagenesis (Agilent Technologies, San Diego, CA, USA). All PCR derived constructs were verified by sequencing.

### Cell Cultures and Transfections

Human embryonic kidney (HEK) 293A cells (Life Technologies, Carlsbad, CA, USA) were grown in Dulbecco’s modified Eagle medium with 10% fetal bovine serum. Chinese hamster ovary (CHO) hm1 cells [20] were grown in alpha minimum essential medium with 5% fetal bovine serum and 500 µg/ml G418 sulfate. TransIT-LT1 reagent (Mirus Bio, Madison, WI, USA) and expression plasmids were used for transient transfections.

### Immunoprecipitation and *In Vitro* Binding Assay

HEK293A cells were co-transfected with FLAG-tagged KCNQ2 and V5-epitope tagged CaM, and were used for immunoprecipitation as described [17]. Briefly, 36 h following transfection, the cells were harvested and lysed in 500 µL HTE buffer containing calcium or calcium chelating reagents according to the experimental condition (150 mM NaCl, 20 mM HEPES (pH 7.4), 50 mM NaF, 1 mM Na_3_VO_4_, 1% Triton X-100 and 5 mM EDTA + 5 mM EGTA or 100 µM CaCl_2_ and complete protease inhibitor cocktail (Roche Applied Science)). After centrifugation at 18,000 x g for 15 min, the supernatants were further precleared by incubation with protein G resin. KCNQ2-FLAG protein was purified using 10 µL anti-FLAG antibody-conjugated resin. Following incubation at 4°C, the immunoprecipitates were washed with 750 µL of corresponding HTE buffer. Protein binding was analyzed by SDS–PAGE and immunoblotting with HRP-conjugated anti-FLAG and anti-V5 antibodies. For the AKAP150 *in vitro* binding assay, AKAP150-His6 protein was purified from *E. coli* BL21 (DE3) using Ni-NTA resin. Protein purification was confirmed by Coomassie staining. 1 µg AKAP150-His6 protein was added to the HEK293A cell lysate transiently expressing KCNQ2-FLAG and CaM-V5 immediately after the preclearance step, and followed by the immunoprecipitation procedure described above. KCNQ2 binding proteins were analyzed by immunoblotting with anti-AKAP150 antibody, anti-V5 and anti-FLAG antibodies. Data were quantified with ImageJ software (NIH).

### Electrophysiological Measurements

Patch-clamp recordings were performed at room temperature on isolated CHO cells using an Axopatch 200B patch-clamp amplifier (Molecular Devices, Sunnyvale, CA, USA) as described [17]. Series resistance compensation was set to 75–86%. Signals were sampled at 2 kHz, filtered at 1 kHz, and acquired using pCLAMP software (Version 7, Molecular Devices). For Ci-VSP experiments, sampling frequency was 500 Hz. Cells were perfused with a solution containing 144 mM NaCl, 5 mM KCl, 2 mM CaCl_2_, 0.5 mM MgCl_2_, 10 mM glucose and 10 mM HEPES (pH 7.4). Patch pipettes (3 – 4 MΩ) were filled with intracellular solution containing 135 mM potassium aspartate, 2 mM MgCl_2_, 1 mM EGTA, 0.1 mM CaCl_2_, 4 mM ATP, 0.1 mM GTP and 10 mM HEPES (pH 7.2). Other EGTA concentrations were used as indicated otherwise. Cells were held at –70 mV and KCNQ2 channels were activated by two-step test pulses to 0 mV followed by –60 mV, with 500 ms duration for each step. KCNQ2 currents were measured at the end of the 0 mV step. To obtain a voltage-current relationship in Ci-VSP experiments, cells were held at –70 mV and 10 s step depolarizations were applied in 10 mV steps from –10 to +40 mV with 2 min inter-step intervals to allow PIP2 regeneration. To measure calcium effect on PIP2 affinity, 1 µM ionomycin was applied one minute following the 10 s step depolarization to +10 mV, and another trace was recorded at +10 mV one minute later. In control experiments, current decay was measured with two 10-s step depolarizations to +10 mV recorded with 2-minute intervals. P-values for statistical analyses were calculated using Prism 6 (GraphPad software, La Jolla, CA, USA) and Excel (Microsoft, Redmond, WA, USA).

### Live Cell Imaging

Transiently transfected CHO cells were plated onto 18-mm glass coverslips 24 hours after transfection. TIRF signals were measured as described previously [17], [20]. Briefly, images were acquired with an inverted microscope IX-81 (Olympus, Tokyo, Japan) and an ImageEM CCD camera (Hamamatsu Photonics, Shizuoka, Japan), and processed with MetaMorph 7.6.3 (Molecular Devices, Sunnyvale, CA, USA). For TIRF experiments, the excitation source was a 445-nm diode laser (Coherent, Santa Clara, CA, USA) with an acousto-optical tunable filter. Low light intensity was used to minimize photobleaching. Images were collected every 10 s with an exposure time of 100 ms.

## Results

**Figure 1:**
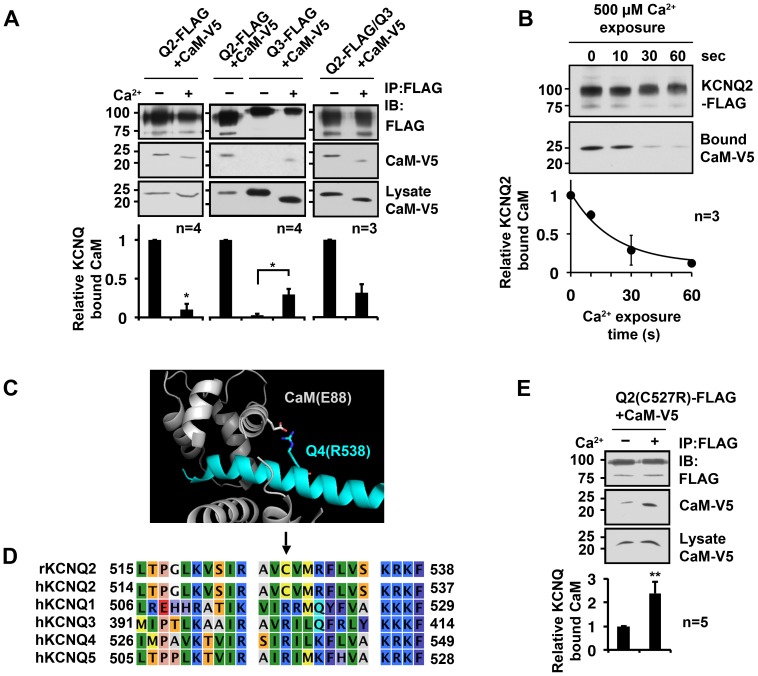
Interaction of CaM with KCNQ subunits required distinct calcium conditions. **A**, Distinct calcium requirement for CaM binding to the KCNQ2, KCNQ3 and KCNQ2/KCNQ3 channels. Representative immunoblots (top) of immunoprecipitation experiments and summary histogram (bottom) are shown. **B,** Time course of CaM dissociation from the KCNQ2 subunit upon exposure to 500 µM Ca^2+^. **C,** Molecular structure from the PDB file (4GOW) depicting the interaction between calcium-bound CaM(E88) and KCNQ4(R538). **D,** Sequence alignment of Helix B, the distal CaM binding domain, of KCNQ1-5 subunits. Arrow shows the cysteine residue on KCNQ2, rKCNQ2(C527), that corresponds to the conserved arginine residue in other KCNQ subtypes, such as KCNQ4(R538); r – rat, h – human. **E,** KCNQ2(C527R) mutation regained CaM binding in the presence of Ca^2+^. * < 0.05, ** < 0.01 by Mann-Whitney test. Error bars show S.E.

Whether calcium disturbs the interaction between calmodulin (CaM) and KCNQ channels has been controversial among several labs [4], [5], [8], [11]. To address this issue, we compared KCNQ co-immunoprecipitated CaM from transiently transfected HEK293A cells in calcium (+) and calcium (–) immunoprecipitation buffers. In the KCNQ2 channel immunoprecipitates, we detected a reduced amount of CaM in the presence of 100 µM calcium (Fig. 1A). In contrast, immunoprecipitation of homomeric rat KCNQ3 channel protein indicated facilitated CaM binding in the presence of calcium (Fig. 1A). However, the overall signal of CaM that co-precipitated with the FLAG-tagged KCNQ3 channel was much weaker than that of the FLAG-tagged KCNQ2 co-precipitate in either calcium (+) or calcium (–) conditions. When heteromeric KCNQ2/KCNQ3 channels were used for immunoprecipitation, calcium reduced co-immunoprecipitated CaM (Fig. 1A). However, the overall retention of CaM in the presence of calcium was increased in heteromeric KCNQ2/KCNQ3 channels compared to homomeric KCNQ2 channels. These results suggest that holoCaM shows distinct binding profiles among KCNQ subtypes.

To further characterize the effect of calcium on the interaction between CaM and KCNQ2 channel, we examined the time course of calcium-induced dissociation of CaM from KCNQ2 protein (Fig. 1B). After the KCNQ2-CaM complex was purified by immunoprecipitation in calcium (–) condition, dissociation of CaM from KCNQ2 protein was examined by exposing KCNQ2-CaM resin to calcium (Fig. 1B). The observed decrease in CaM co-immunoprecipitation occurred rather slowly, with an estimated time constant of 20.7±3.5 s (n  =  3). This is much slower than the calcium-induced conformational change of CaM that has been shown to have micro- to milli-second scale kinetics [21].

Recently, the crystal structure of a protein complex formed by hKCNQ4 and holoCaM has been reported [11]. The solved structure indicates that holoCaM stably binds to Helix B of the channel protein. In contrast, stable association with apoCaM requires two CaM binding sites on the KCNQ2 channel, Helix A and Helix B [4], [5]. The reported holoCaM-KCNQ4 channel structure indicates a salt bridge between R538 of hKCNQ4 channel and E88 of CaM, which caught our attention (Fig. 1C). Interestingly, this arginine residue in the KCNQ4 channel, R538, is conserved in all KCNQ subtypes except KCNQ2. In the corresponding position in rat KCNQ2, the channel contains cysteine, KCNQ2(C527) (Fig. 1D). Therefore, we suspected that the arginine residue at this position determined the stable binding of holoCaM. To test this idea, we examined CaM binding to the KCNQ2(C527R) mutant channel. CaM was able to bind KCNQ2(C527R) protein in the calcium (–) condition (Fig. 1E). However, in contrast to the wild-type KCNQ2 subunit, addition of 100 µM calcium further increased binding of CaM to KCNQ2(C527R) (Fig. 1E). These results suggest that the conserved arginine residue at this position plays a critical role in holoCaM retention.

**Figure 2:**
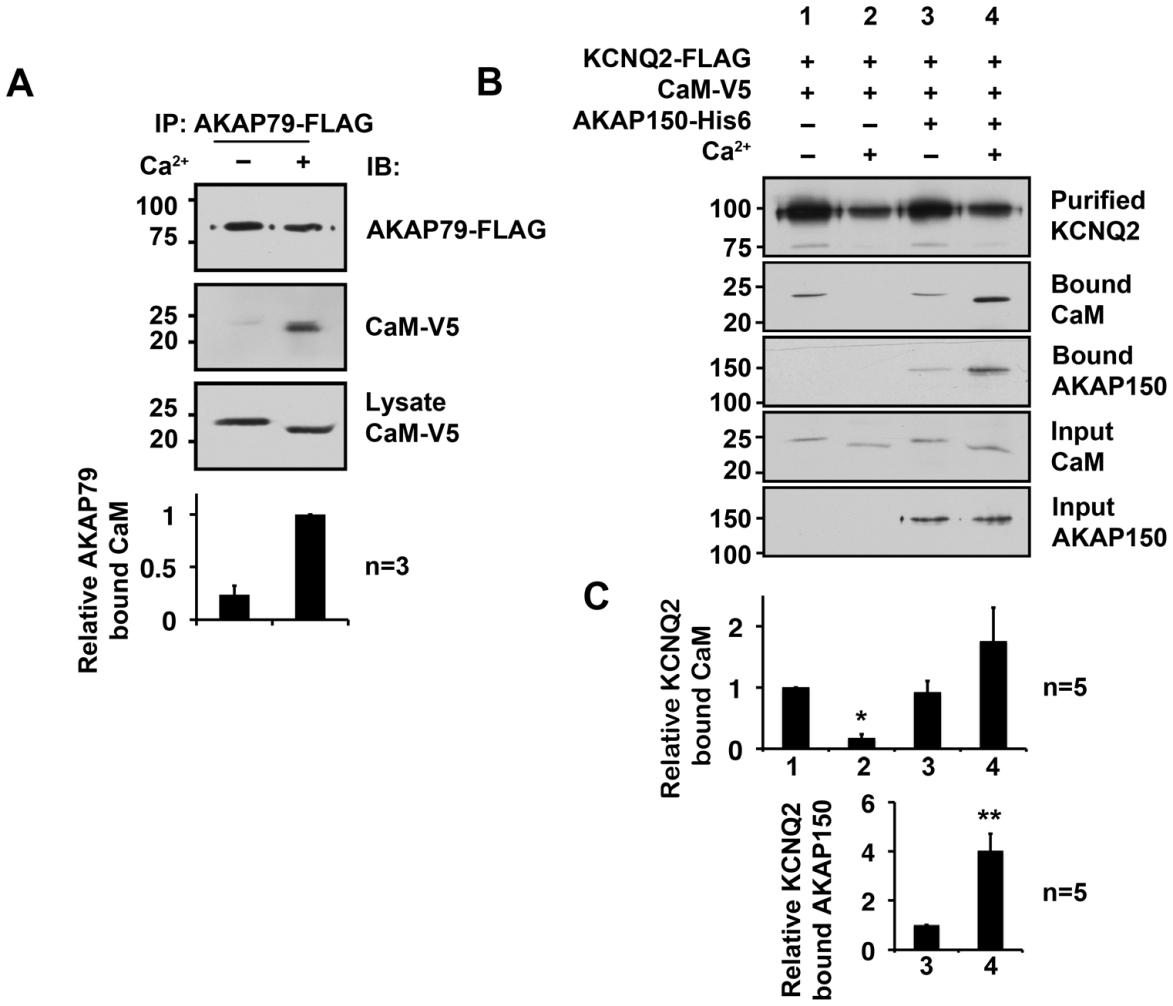
HoloCaM was retained in the KCNQ2 channel complex via AKAP150. **A,** Calcium (100 µM) dependent binding of CaM to AKAP79. **B,** *In vitro* binding of KCNQ2 and CaM with or without AKAP150. KCNQ2-FLAG was immunopurified by anti-FLAG conjugated resin. CaM was co-purified in calcium (+) condition only in the presence of AKAP150. **C,** Top – the summary of quantification of relative KCNQ2-bound CaM from five independent experiments shown in B**.** Bottom – the summary of quantification of relative KCNQ2-bound AKAP150 from five independent experiments shown in B. Bars are labeled corresponding to the lane numbers on the immunoblot. *<0.05 non-parametric ANOVA followed by Dunn’s multiple comparisons test, ** < 0.01 by Mann-Whitney test. Error bars show S.E.

KCNQ2 subunit associates with another CaM binding protein as a constituent of the channel complex: AKAP79/150, a scaffold protein critical for KCNQ2 channel regulation [18], [22]. It has been demonstrated that AKAP79/150 binds holoCaM but not apoCaM [23], [24]. Our immunoprecipitation experiments confirmed that CaM binds to AKAP79 only in the presence of calcium (Fig. 2A). To test the overall effect of the AKAP150/KCNQ2 channel complex on CaM retention, we conducted *in vitro* binding experiments using CaM and KCNQ2 proteins with or without AKAP150 protein (Fig. 2B). In control experiments in the absence of AKAP150, the binding of holoCaM to KCNQ2 protein was significantly lower than that of apoCaM, as shown in Fig. 1 & 2B. However, when AKAP150 was present, holoCaM was retained in the KCNQ2 channel complex (Fig. 2B & 2C). In addition, the binding between KCNQ2 and AKAP150 was also augmented in the presence of calcium, which may have contributed to an increase in CaM retention (Fig. 2C). Collectively, our *in vitro* binding and immunoprecipitation data suggest that holoCaM dissociated from the KCNQ2 subunit. However, AKAP150 could stabilize the KCNQ2 channel complex in the presence of calcium, as evidenced by strong calmodulin binding in the immunoprecipitates containing AKAP150. Alternatively, the dissociation of holoCaM from the KCNQ2 subunit could be attenuated by the KCNQ3 subunit.

**Figure 3:**
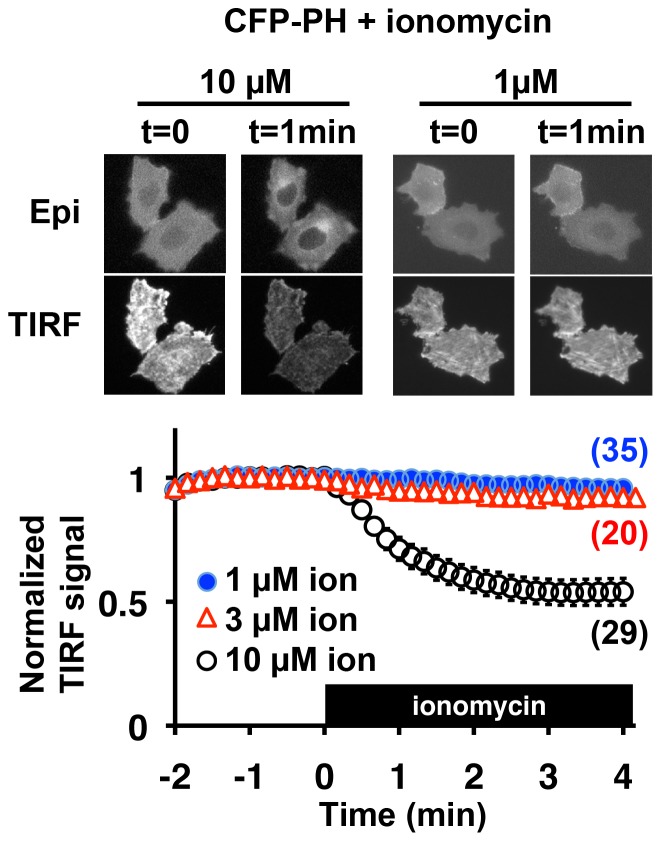
Ionomycin treatment and PIP2 depletion. TIRF analysis indicating membrane localization of the CFP-PH probe. Top panels show epifluorescent cell images (epi) indicating total fluorescence, and TIRF images (TIRF) showing plasma membrane localization. The lower panel shows pooled data from TIRF analyses. 10 µM ionomycin induced translocation of CFP-PH, which indicates depletion of PIP2. In contrast, 1 µM or 3µM ionomycin did not alter plasma membrane localization of CFP-PH. TIRF signal is normalized to that at t  =  0. Black box indicates the presence of ionomycin. Error bars show S.E.

**Figure 4:**
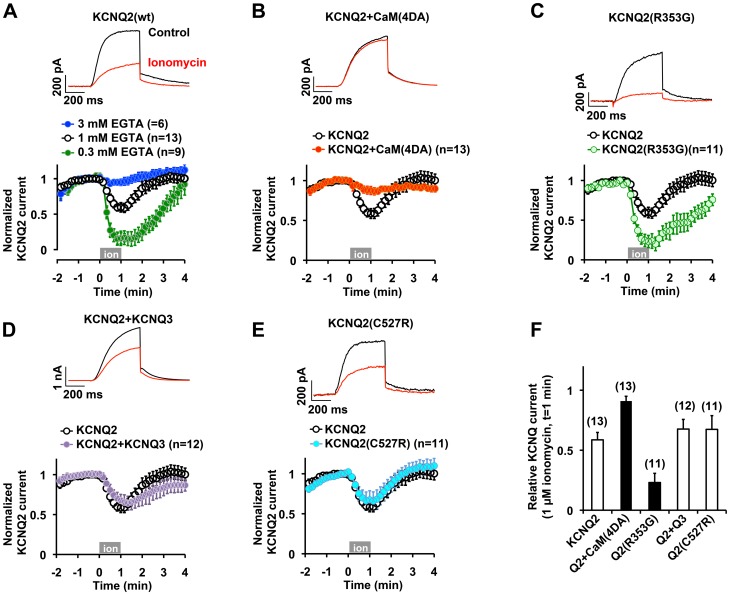
Ionomycin suppressed KCNQ currents via elevation of intracellular calcium. **A,** A representative KCNQ2 current response to 1 µM ionomycin with 1 mM EGTA in the pipette solution. The lower panel summarizes ionomycin-induced suppression of the KCNQ2 current, which was dependent on EGTA concentration in the pipette solution. Gray box indicates the presence of 1 µM ionomycin. 1 mM EGTA was used for the rest of the experiments. **B,** Ionomycin-induced KCNQ2 current suppression was prevented by co-expression of a dominant negative CaM, CaM(4DA). Control ionomycin response of KCNQ2 channel from panel A is also shown. **C,** Ionomycin induced augmented suppression of the KCNQ2(R353G) current. KCNQ2 response from panel A is also shown. **D,** Ionomycin response of the heteromeric KCNQ2/KCNQ3 channels was similar to that of the homomeric KCNQ2 channels. **E,** Ionomycin response of the KCNQ2(C527) channel. **F,** Summary of ionomycin responses. Relative KCNQ current at t  =  1 min is shown. Black bars indicate responses significantly different (p < 0.01, nonparametric ANOVA followed by Dunnett’s multiple comparisons test) from the wild-type KCNQ2 response. Error bars show S.E.

Our results indicate that KCNQ3 and KCNQ2(C527R) channels showed drastic differences in regards to holoCaM binding compared to the wild-type KCNQ2. We next focused on the functional consequences of such a change in holoCaM binding. We selected ionomycin, a calcium ionophore, as a means of elevating intracellular calcium levels since it would not require the activation of cellular signaling mechanisms to raise calcium. However, it has been shown that ionomycin can activate phospholipase C δ (PLCδ) [25], [26], which would deplete PIP2 and suppress KCNQ current. To evaluate ionomycin effects on PLCδ in our experimental conditions, PIP2 levels in CHO cells were assessed by a PIP2 probe carrying the PIP2 binding site of PLCδ and CFP, CFP-PH [12], [25], [26]. Plasma membrane localization of CFP-PH was monitored by TIRF microscopy. Indeed, 10 µM ionomycin induced PIP2 depletion as indicated by the translocation of CFP-PH as described [25], [26] (Fig. 3). However, we found that ionomycin concentrations of up to 3 µM did not alter plasma membrane localization of CFP-PH (Fig. 3). Thus, we examined the functional effect of 1 µM ionomycin on the KCNQ2 current. Application of 1 µM ionomycin induced KCNQ2 current suppression. The level of current suppression was dependent on the concentration of EGTA in the patch pipette solution (Fig. 4A), confirming that the suppression was due to the rise in intracellular calcium levels. In addition, this calcium-induced KCNQ2 current suppression was prevented by co-expression of a dominant negative CaM, CaM(4DA), which contains alanine mutations in all four EF hand motifs, as reported by Gamper et al. [8] (Fig. 4B). These data indicate that the increase in intracellular calcium sensed by CaM was responsible for the KCNQ2 current suppression.

Since holoCaM gradually dissociated from the KCNQ2 channel as shown in Fig. 1, we questioned whether a KCNQ2 mutant with impaired CaM binding would be more susceptible to ionomycin stimulation. To test this, we examined the KCNQ2(R353G) mutant. This mutation causes impaired CaM binding and is found in epilepsy patients [6], [17]. As expected, application of 1 µM ionomycin resulted in augmented and almost complete suppression of the KCNQ2(R353G) current (Fig. 4C). These results support the hypothesis that changes in calcium levels alter KCNQ2-CaM binding, which leads to the suppression of the current.

As we showed in Fig. 1, the KCNQ3 subunit showed facilitated binding to holoCaM, which suggests a change in the configuration of protein-protein interaction. Thus, we examined whether the change in CaM binding configuration rather than its dissociation from the channel was sufficient to modulate the current. We measured responses of heteromeric KCNQ2/KCNQ3 and homomeric KCNQ2(C527R) channels to ionomycin treatment. Interestingly, even though the KCNQ2(C527R) mutation resulted in a drastic change in CaM binding, KCNQ2(C527) channels showed normal functional expression at basal conditions (25.4±4.7 pA/pF at 500 ms, 0 mV, n  =  11, P>0.05). In addition, both KCNQ2/3 and KCNQ2(C527R) channels showed equivalent ionomycin-induced suppression compared to the homomeric wild-type KCNQ2 channel (Fig. 4D, E and F). These results suggest that the calcium-induced change in CaM binding configuration was sufficient for the KCNQ2 current suppression.

**Figure 5:**
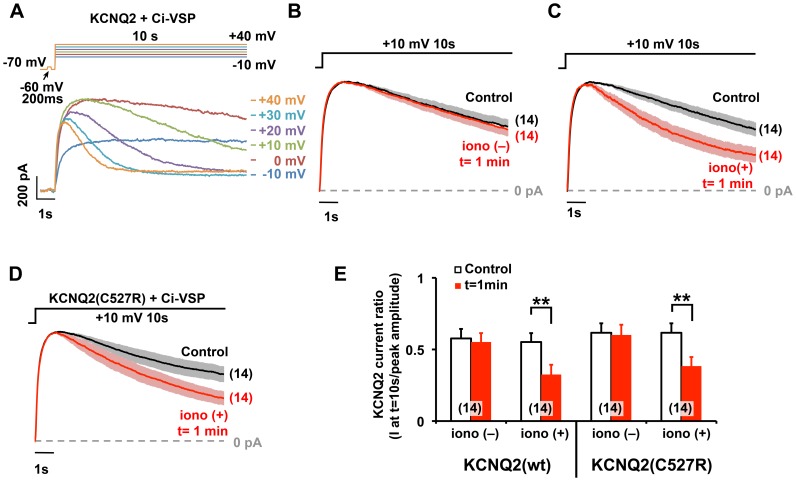
Ionomycin-induced suppression of KCNQ2 current was accompanied by a lower PIP2 affinity. **A,** Representative current traces showing a voltage dependent KCNQ2 current decay due to the activation of Ci-VSP. A brief voltage step to –60 mV was applied to calculate the linear leak. **B,** Scaled KCNQ2 current traces at +10 mV showing an identical Ci-VSP-mediated current decay without ionomycin at 2-min interval. Gray and shaded red areas show S.E. **C,** Scaled KCNQ2 current traces showing the facilitation of KCNQ2 current decay 1 min after the application of 1 µM ionomycin. Control traces were obtained 1 min before ionomycin application. **D,** Ci-VSP-mediated current decay of the KCNQ2(C527R) current. Scaled current traces indicate the facilitation of current decay by 1 µM ionomycin at +10 mV. **E,** Summary of the Ci-VSP-induced current decay at control (t  =  –1 min) and t  =  1 min for indicated conditions. KCNQ2(wt) and KCNQ2(C527R) showed an equivalent Ci-VSP-mediated current decay both with and without 1µM ionomycin. **<0.01 by paired t-test. Error bars show S.E.

We then asked how holoCaM suppressed the KCNQ2 current. We have previously demonstrated that PKC-mediated dissociation of CaM from the KCNQ2 channel lowers affinity to PIP2, which results in current suppression [17]. We suspected that a calcium-induced change in CaM binding configuration may also lower the PIP2 affinity of KCNQ channels. To test this, a voltage-sensitive phosphatase, Ci-VSP, which catalyzes PIP2 [19], was co-expressed with the KCNQ2 channel. Activation of Ci-VSP has been demonstrated to deplete PIP2 and suppress KCNQ2 currents upon depolarization to voltages more positive than 0 mV [27], [28]. Since the KCNQ2 channel *per se* does not inactivate, we measured the depolarization-induced current decay caused by the Ci-VSP-mediated PIP2 depletion. In the cells co-expressing Ci-VSP and KCNQ2, KCNQ2 currents exerted a current decay during the 10-s depolarization step at voltages more positive than 0 mV (Fig. 5A). We then compared current decay at +10 mV in the control condition and after 1 µM ionomycin application (Fig. 5B, C, E). Ionomycin treatment further facilitated Ci-VSP-mediated KCNQ2 current decay (Fig. 5C), suggesting that after calcium exposure, KCNQ2 channels were more susceptible to PIP2 depletion. Next, we examined the effect of Ci-VSP on the KCNQ2(C527R) channel, in which CaM binding was facilitated by calcium (Fig. 5D). When co-expressed with Ci-VSP, a 10-s depolarization step to +10 mV induced a current decay, which was equivalent to that of the wild-type KCNQ2 channel at basal conditions (Fig. 5E). The application of ionomycin further facilitated the current decay to an extent comparable to that of the wild-type KCNQ2 current (Fig. 5D and E). These results suggest that a change in the configuration of CaM–KCNQ2 binding induced by elevated intracellular calcium led to the reduction in PIP2 affinity of the KCNQ2 channel. Furthermore, this Ci-VSP-mediated current rundown occurred in both wild-type and KCNQ2(C527R) channels, despite the observed differences in calcium preference of the two constructs for CaM interaction (Fig. 5E). These results suggest that the change in CaM binding configuration rather than CaM dissociation from the KCNQ channel was key for this modulation.

## Discussion

In this report, we demonstrated that calcium-mediated KCNQ current suppression involves CaM and the reduction in the affinity of the KCNQ2 channel for PIP2. We confirmed that CaM is the calcium sensor for the KCNQ2 channel as previously described [8]. We also demonstrated that a CaM deficient mutant channel, KCNQ2(R353G), showed augmented ionomycin-induced current suppression, which suggests that a change in the KCNQ2-CaM interaction is critical for the suppression. Finally, we demonstrated that ionomycin increased the vulnerability of KCNQ2 channels to PIP2 depletion.

Whether holoCaM can bind KCNQ subunits has been controversial [4], [5], [8], [11]. We determined that C527 of KCNQ2, which corresponds to an arginine residue in other KCNQ subunits, was responsible for the dissociation of holoCaM from KCNQ2. Accordingly, we were able to demonstrate that holoCaM binding was maintained by the KCNQ2(C527R) mutant channels. We also demonstrated that various factors, such as the presence of KCNQ3 subunit or AKAP79/150 in the channel complex could alter holoCaM binding. In our experimental conditions, heteromeric KCNQ2/KCNQ3 channels showed reduced CaM binding in the presence of calcium. However, since different stoichiometry of subunit composition could result in inconsistent holoCaM retention, the use of heteromeric KCNQ2/KCNQ3 channels to study CaM interaction may have caused the conflicting results in the past [4], [5], [29].

We have previously demonstrated that the dissociation of CaM from the KCNQ2 channel induced by PKC phosphorylation of serine 541 on KCNQ2 results in current suppression [17]. The current study suggests that not only the calcium-induced dissociation of CaM, but also a change in the configuration of CaM-KCNQ2 binding could suppress the KCNQ2 current. In fact, even though KCNQ2 and KCNQ3 channels showed distinct calcium dependent CaM binding, heteromeric KCNQ2/KCNQ3 channels showed equivalent responses to ionomycin treatment. Similarly, despite the facilitated holoCaM binding by the KCNQ2(C527R) mutant, KCNQ2(C527R) currents showed ionomycin-induced suppression that was comparable to that of the wild-type KCNQ2 currents. The observation that the KCNQ2(C527R) mutant channel had normal channel function at the basal conditions, while showing distinct holoCaM binding, suggests that the arginine residue in Helix B interacts with holoCaM but not apoCaM, which would support the recent crystal structure [11] as a physiological configuration. In addition, since apoCaM requires both Helix A and Helix B for stable binding, loss of contact with Helix A by holoCaM could be responsible for the reduced KCNQ affinity toward PIP2.

We demonstrated that ionomycin increased the susceptibility of the channel to PIP2 depletion induced by Ci-VSP. This suggests that ionomycin reduced the affinity of the KCNQ2 channel for PIP2. Since the KCNQ2(R353G) channel with impaired CaM binding and low PIP2 affinity [17] showed a stronger response to ionomycin, it would be reasonable to assume that the KCNQ2-CaM interaction regulates PIP2 affinity of the KCNQ2 subunit as we reported previously [17]. However, since calcium can activate other signaling cascades, we cannot deny that other mechanisms may contribute to the reduction of KCNQ2 affinity toward PIP2. For example, phosphorylation of the KCNQ2 subunit in response to calcium rise or the overall rearrangement of the protein complex could cause a decrease in PIP2 affinity. Indeed, a large increase in intracellular calcium would activate the PLCδ pathway and further amplify calcium-induced KCNQ current responses. In physiological conditions, it is likely that a combination of the PLCδ pathway and the reduction in PIP2 affinity synergistically suppress the KCNQ2 current, similarly to the muscarinic KCNQ2 current suppression via PIP2 depletion and PKC-mediated reduction in PIP2 affinity [11].

In conclusion, signaling events that can change the configuration of CaM-KCNQ2 binding, such as elevation of intracellular calcium or PKC phosphorylation, lower KCNQ2 affinity toward PIP2. We propose that CaM is the molecular switch for controlling PIP2 affinity of the KCNQ channels.
